# Effect of *N*-Terminal Peptide Modifications on In Vitro and In Vivo Properties of ^177^Lu-Labeled Peptide Analogs Targeting CCK2R

**DOI:** 10.3390/pharmaceutics15030796

**Published:** 2023-02-28

**Authors:** Anton Amadeus Hörmann, Maximilian Klingler, Christine Rangger, Christian Mair, Lieke Joosten, Gerben M. Franssen, Peter Laverman, Elisabeth von Guggenberg

**Affiliations:** 1 Department of Nuclear Medicine, Medical University of Innsbruck, 6020 Innsbruck, Austria; 2 Department of Medical Imaging, Nuclear Medicine, Radboud University Medical Center, 6525 GA Nijmegen, The Netherlands

**Keywords:** cholecystokinin-2 receptor, gastrin, peptide receptor radionuclide therapy, lutetium-177, theranostics

## Abstract

The therapeutic potential of minigastrin (MG) analogs for the treatment of cholecystokinin-2 receptor (CCK2R)-expressing cancers is limited by poor in vivo stability or unfavorable accumulation in non-target tissues. Increased stability against metabolic degradation was achieved by modifying the C-terminal receptor-specific region. This modification led to significantly improved tumor targeting properties. In this study, further *N*-terminal peptide modifications were investigated. Two novel MG analogs were designed starting from the amino acid sequence of DOTA-MGS5 (DOTA-DGlu-Ala-Tyr-Gly-Trp-(*N*-Me)Nle-Asp-1Nal-NH_2_). Introduction of a penta-DGlu moiety and replacement of the four *N*-terminal amino acids by a non-charged hydrophilic linker was investigated. Retained receptor binding was confirmed using two CCK2R-expressing cell lines. The effect on metabolic degradation of the new ^177^Lu-labeled peptides was studied in human serum in vitro, as well as in BALB/c mice in vivo. The tumor targeting properties of the radiolabeled peptides were assessed using BALB/c nude mice bearing receptor-positive and receptor-negative tumor xenografts. Both novel MG analogs were found to have strong receptor binding, enhanced stability, and high tumor uptake. Replacement of the four *N*-terminal amino acids by a non-charged hydrophilic linker lowered the absorption in the dose-limiting organs, whereas introduction of the penta-DGlu moiety increased uptake in renal tissue.

## 1. Introduction

The importance of radiolabeled peptides used in therapeutic intervention and diagnosis of tumor malignancies is rapidly increasing in modern medicine. These radiopeptides specifically recognize and bind to specific receptors on the cell surface. The cholecystokinin-2-receptor (CCK2R) belongs to a family of G-protein-coupled receptors found in the central nervous system and in the gastrointestinal tract. The receptor plays a role in modulating anxious behavior in the brain, as well as secreting gastric acid in the stomach [1]. Several malignant tumors, including medullary thyroid carcinoma, small cell lung cancer, astrocytoma, stromal ovarian cancer, gastrointestinal stromal tumors, leiomyosarcoma, as well as some gastrointestinal neuroendocrine tumors, breast, and endometrial adenocarcinomas [2,3], overexpress this receptor. Many efforts have been made in the past to develop a targeted therapy using radiolabeled peptide derivatives of gastrin and cholecystokinin [4,5,6,7]. The use of radiopeptides in peptide receptor radionuclide therapy (PRRT) is coupled to several prerequisites, such as sufficient in vivo stability, high accumulation in tumor tissue with concomitant low uptake in non-target tissue, and predominant excretion via the renal system [8]. Early development of radioiodinated gastrin analogs demonstrated the feasibility of CCK2R targeting for therapeutic applications [9]. Therapeutic isotopes based on radiometals were introduced soon thereafter. The straightforward complexation chemistry based on bifunctional chelators, simplified the preparation of the radiopeptides for clinical use. Minigastrin (MG, Leu-(Glu)_5_-Ala-Tyr-Gly-Trp-Met-Asp-Phe-NH_2_), a member of the gastrin peptide hormone family, shares the *C*-terminal binding sequence “Trp-Met-Asp-Phe-NH_2_”, which is critical for receptor affinity [1,10,11]. This amino acid sequence was conjugated to the acyclic chelator diethylenetriaminepentaacetic acid (DTPA). In addition, leucine in position 1 was replaced by D-glutamic acid (DGlu) to improve the thermodynamic stability and kinetic inertness of the complex [12]. DTPA-DGlu^1^-minigastrin (DTPA-MG0, DTPA-DGlu-(Glu)_5_-Ala-Tyr-Gly-Trp-Met-Asp-Phe-NH_2_) could be stably radiolabeled with β-minus emitting particles or γ-emitting radionuclides, such as yttrium-90 or indium-111 [12,13]. Nevertheless, therapeutic implementation in the clinic was rather limited, since a major drawback was the high renal uptake that caused unwanted side effects [14]. The penta-Glu moiety was linked to enhanced renal absorption. This moiety also had a significant impact on in vivo stability. For the truncated MG analog conjugated to the macrocyclic chelator 1,4,7,10-tetraazacyclododecane-1,4,7,10-tetraacetic acid (DOTA-MG11, DOTA-DGlu-Ala-Tyr-Gly-Trp-Met-Asp-Phe-NH_2_), an inferior enzymatic stability was observed [14,15,16]. In a multicenter study funded by the European Cooperation in Science and Technology (COST BM0607: targeted radionuclide therapy), twelve novel peptide analogs comprising the introduction of natural/unnatural amino acids, cyclization, or dimerization, mainly in the *N*-terminal part of the linear peptide, were investigated [4,5,7]. DOTA-PP-F11 (DOTA-(DGlu)_6_-Ala-Tyr-Gly-Trp-Met-Asp-Phe-NH_2_), with the penta-Glu moiety switched to the D-isomeric form, showed favorable biodistribution with low kidney retention in female BALB/c mice. However, the stability issues were not considerably improved [4]. Site-specific exchange of amino acids in the *C*-terminal receptor specific binding sequence led to new peptide analogs with high stability against metabolic digestion in vivo. By the replacement of phenylalanine with 1-naphtylalanine (1Nal), as well as the substitution of oxidation-sensitive methionine for *N*-methylated norleucine ((*N*-Me)Nle), the new MG analog DOTA-MGS5 (DOTA-DGlu-Ala-Tyr-Gly-Trp-(*N*-Me)Nle-Asp-1Nal-NH_2_) was developed. In comparison with previously developed MG analogs, this peptide derivative radiolabeled with various radiometals exhibited enhanced receptor-specific cellular internalization and high resistance to enzymatic degradation leading to superior tumor targeting properties in vivo [17].

For DOTA-MGS5 labeled with lutetium-177, 86% and 70% intact radiopeptide was detected in the blood of BALB/c mice 10 min and 1 h after intravenous injection, respectively [17]. Further introduction of proline in different positions close to the *N*-terminus did not further improve the stability in vivo [18]. In the attempt to investigate possible alternative stabilization strategies in this study, the effect of the *N*-terminal modifications within the peptide sequence on the in vitro and in vivo CCK2R-targeting properties was evaluated. Based on [^177^Lu]Lu-PP-F11N, which is currently investigated in clinical studies (ClinicalTrials.gov Identifier: NCT02088645), the amino acid sequence of DOTA-MGS5 was modified by introducing a penta-DGlu moiety [19]. Using a combination of the amino acid sequence of pentagastrin (PG, BOC-βAla-Trp-Met-Asp-Phe-NH_2_) and the *C*-terminal modifications of DOTA-MGS5, the four *N*-terminal amino acids were replaced by a non-charged hydrophilic linker. Two moieties of 4-amino-3-hydroxybutyric acid (GABOB) and one residue of beta-alanine (βAla) were introduced as a spatial distance between the chelator and the receptor-specific amino acid sequence. Direct conjugation of the chelator to the pharmacophore region of the peptide was avoided to not interfere with CCK2R affinity. Reduced receptor affinity was found for des-BOC-pentagastrin (βAla-Trp-Met-Asp-Phe-NH_2_) and CCK4 (Trp-Met-Asp-Phe-NH_2_), radioiodinated using the Bolton–Hunter reagent [10]. Similar findings were obtained with CCK4 directly conjugated to a bifunctional chelator, whereas the introduction of βAla and 6-aminohexanoic acid (Ahx) linkers allowed to retain affinity [20,21]. The in vitro characteristics of the new ^177^Lu-labeled peptide analogs were investigated, with specific focus on the stability against enzymatic degradation. Receptor affinity of the peptide analogs, as well as cell internalization of the radiolabeled conjugates was studied using A431 epidermoid carcinoma cells stably transfected to express the human CCK2R (A431-CCK2R), as well as AR42J rat pancreatic cells expressing rat CCK2R [22]. Metabolic studies in BALB/c mice were performed to confirm a high resistance of the radiolabeled peptides against metabolic degradation also in vivo. The biodistribution and tumor targeting properties were evaluated in A431-CCK2R xenografted BALB/c nude mice, including dosimetry estimates for dose-limiting organs.

## 2. Materials and Methods

### 2.1. Materials 

All chemicals used were of analytical quality and commercially available. The reagents were not further purified. Isotope Technologies Munich supplied non-carrier-added [^177^Lu]LuCl_3_ (ITM, Garching, Germany). Dr. Luigi Aloj contributed the A431-CCK2R cells transfected to stably express human CCK2R as well as A431-mock cells transfected with the empty vector [23]. The AR42J rat pancreatic cells physiologically expressing rat CCK2R were obtained via ECACC (Salisbury, UK). Dulbecco’s Modified Eagle Medium (DMEM) was used to cultivate both A431-cell lines, whereas RPMI 1640 medium was used to cultivate the AR42J cells. Ten % (*v*/*v*) fetal bovine serum and 5 mL of a 100x penicillin-streptomycin-glutamine solution were added to the cell culture medium. Cells were then grown at 37 °C in a humidified 95% air/5% CO_2_ environment. Trypsin/EDTA solution was used to collect the cells (Sigma-Aldrich, Steinheim, Germany). Invitrogen Corporation supplied the media and supplements (Lofer, Austria). The peptide analog DOTA-MGS5 was provided by piCHEM (Raaba-Grambach, Austria).

### 2.2. Peptide Synthesis

The new minigastrin analogs DOTA-[(*N*-Me)Nle^11^,1Nal^13^]PP-F11N (**1**) and DOTA-[(GABOB)_2_,desBOC,(*N*-Me)Nle^3^,1Nal^5^]]-PG (**2**) were synthesized by standard solid phase peptide synthesis using 9-fluorenylmethoxycarbonyl (Fmoc)-protected amino acids as described previously [24]. The following protective groups were used to protect the amino acids’ reactive side chains: tert-butyl ester for Asp and DGlu, tert-butyl ether for Tyr, and tert-butyloxycarbonyl (BOC) for Trp. For coupling tris(tert-butyl) protected DOTA, a 3-fold molar excess was used.

Purification was carried out using RP-HPLC on a GILSON UV/VIS-155D multi-wavelength UV detector, equipped with an Eurospher II 100 Å 5 µm C18 column, 250 mm × 8 mm (Knauer, Berlin, Germany), combined with an Eurosil Bioselect 300 Å 5 µm C18 precolumn, Vertex Plus A, 30 mm × 8 mm (Knauer, Berlin, Germany), using a water/0.1% TFA (A) and acetonitrile/0.1% TFA (B) gradient with a flow rate 2 mL/min: 0–4 min 20% B, 4–24 min 20–60% B, 24–26 min 60% B, 26–27 min 60–80% B, 27–28 min 80% B, 28–29 min 80–20% B, and 29–37 min 20% B. The synthesized peptide conjugates with confirmed purity were characterized by MALDI-TOF MS (Bruker Microflex^®^, Bruker Daltonics, Bremen, Germany) lyophilized and stored at −20 °C for further use. Peptides were dissolved in water containing 20% EtOH or PBS (~1 µg/mL).

### 2.3. Radiolabeling and Characterization In Vitro

Radiolabeling with lutetium-177 was carried out using 2–10 µg of DOTA-conjugate, ~50–350 MBq of ≤150 µL [^177^Lu]LuCl_3_ solution and a >1.2-fold volume of a 0.4 M sodium acetate/0.24 M gentisic acid solution with pH 5, reaching a radioactivity concentration of ~0.5–3 GBq/mL. The reaction solution was incubated in a low protein binding tube (Eppendorf AG, Hamburg, Germany) at 90 °C for 20 min. For determination of radiochemical purity (RCP), an UltiMate 3000 chromatography system was used. The system consisted of a variable UV-detector (UV-VIS at λ = 220 nm), an HPLC pump, an autosampler, a radiodetector (GabiStar, Raytest, Straubenhardt, Germany), and was equipped with a Phenomenex Jupiter 4 μm Proteo 90 Å C12 column, 250 mm × 4.6 mm (Phenomenex Ltd., Aschaffenburg, Germany). A flow rate of 1 mL and a water/acetonitrile/0.1% trifluoroacetic acid gradient with increasing concentrations of acetonitrile was used for analysis: (ACN): 0–3 min, 10%; 3–18 min, 10–55%; 18–20 min, 55–80%; 20–21 min, 80–10%; 20–25 min, 10%. Alternatively, an Agilent 1200 System (Agilent Technologies), with an in-line radiodetector (Elysia-Raytest, Liege, Belgium) equipped with a HiChrom C18 5 µm column, 250 mm × 4.6 mm, was used with a flow rate of 1 mL/min and a gradient with increasing concentrations of ACN: 0–5 min, 3%; 5–15 min, 3–100%; 15–25 min, 100%; 25–30 min, 100–3%; 30–35 min, 3%. The absence of radiocolloid formation was confirmed by iTLC-SG with 1 M ammonium acetate and methanol (1/1, *v*/*v*) as mobile phase.

Solid phase extraction (SPE) was used to purify reaction solutions for use in biodistribution experiments. The SepPak^®^ tLight C18 cartridge (Waters, Milford, MA, USA) was pretreated with 5 mL of 99% ethanol and 5 mL of water before loading the reaction solution. To remove hydrophilic impurities, the cartridge was washed with 5 mL of water. Elution of the radiolabeled peptide was performed using 0.7 mL of ethanol and 2.3 mL PBS. The solution for injection was prepared by dilution with PBS containing 0.5% BSA to avoid sticking to the plastic material. The final bolus injection contained 20 pmol of total peptide in ~150 µL with less than 3% EtOH.

The stability studies with the ^177^Lu-labeled peptide analogs were carried out at a concentration of 0.5 nmol peptide/mL in fresh human serum (n = 2) for up to 24 h. A mixture of 0.05 nmol/mL of the radiolabeled peptides in PBS and octanol (1/1, *v*/*v*) was used to evaluate the octanol/PBS distribution coefficient (logD_7.4_; n = 8). To determine the protein binding in human serum by Sephadex G-50 size-exclusion chromatography (GE Healthcare Illustra, Little Chalfont, UK), a 25 µL serum sample was used for different time points after incubation. All steps were performed according to previously published protocols [24].

### 2.4. Cell Uptake and Receptor Binding Studies

Internalization experiments were performed in A431-CCK2R and AR42J cells. The specificity of the cell uptake was confirmed by parallel assays using A431-mock cells without receptor expression. AR42J cells were additionally co-incubated with 1 µM pentagastrin (blocking conditions). The cells were seeded at a density of 1.0 × 10^6^ for A431-CCK2R and 1.5 × 10^6^ per well for the AR42J cells in 6-well plates (Greiner Labortechnik, Kremsmünster, Austria) and grown to confluence for 48 h. The cells were incubated with the different radiopeptides (final peptide concentration of 0.4 nM) at 37 °C for up to 4 h, as described previously [24], and the radioactivity of the lyzed cells was determined in relation to the total radioactivity added (% internalized radioactivity).

The binding affinity for CCK2R of the new peptide analogs was tested in a competition assay against [Leu^15^]gastrin-I radiolabeled with iodine-125 and compared with pentagastrin and DOTA-MGS5 (n = 3). Radioiodination of [Leu^15^]gastrin-I was carried out using the chloramine-T method, as described previously [18]. HPLC purification was used to separate [3-iodo-Tyr^12^,Leu^15^]gastrin-I from non-labeled [Leu^15^]gastrin-I. The radioligand was stored in aliquots of 5 × 10^6^ cpm at −25 °C. Binding experiments were performed using 96-well filter plates (MultiScreenHTS-FB, Merck Group, Darmstadt, Germany) washed with 10 mM TRIS/139 mM NaCl pH 7.4 (2 × 250 µL). For the experiment, 0.4 × 10^6^ A431-CCK2R cells in 100 µL binding buffer (20 mM HEPES buffer with pH 7.4 and 10 mM MgCl_2_, 14 µM bacitracin, and 0.5% BSA) were added to each well. Fifty µL of [^125^I][3-iodo-Tyr^12^,Leu^15^]gastrin-I (~25,000 cpm) and 50 µL of different dilutions of the peptide conjugates (reaching final concentrations of 0.0003–1000 nM) were added and the cells were incubated in triplicates for 1 h at room temperature. Pentagastrin was included as an internal standard. Incubation was interrupted by filtration of the medium and rapid rinsing with ice-cold 10 mM TRIS/139 mM NaCl pH 7.4 (2 × 200 µL). The filters were collected and counted in a γ-counter (2480 Wizard2 3”, PerkinElmer Life Sciences and Analytical Sciences, formerly Wallac Oy, Turku, Finland). Half-maximal inhibitory concentration (IC_50_) values were calculated following nonlinear regression with Origin software (Microcal Origin 6.1, Northampton, MA, USA). For graphical presentation, data of exemplary binding curves were normalized from 0 to 100.

### 2.5. In Vivo Stability

Metabolic and biodistribution studies were performed in accordance with the ethical standards of the institution and approved by the Austrian Ministry of Science (BMWFW-66.011/0072-V/3b/2019).

In vivo stability studies of [^177^Lu]Lu-**1** and [^177^Lu]Lu-**2** were carried out in 5–7-week-old female BALB/c mice (Charles River Laboratories, Sulzfeld, Germany; n = 6). A higher radioactivity of ~37 MBq in a total volume of ~150 µL in PBS/0.5% BSA, corresponding to ~1 nmol total peptide, was administered into the mice intravenously through a lateral tail vein to increase the detectability of potential radiometabolites by radio-HPLC. The mice were euthanized by cervical dislocation after 10 (n = 2) and 30 (n = 1) min post injection and the urine and a venous blood sample were collected at the time of sacrifice. Liver and kidneys were dissected and homogenized in 20 mM HEPES buffer pH 7.3 (1:1, *v*/*v*) with an Ultra-Turrax T8 homogenizer (IKA-Werke, Staufen, Germany) for 1 min at RT. Prior to the radio-HPLC analysis, the samples of blood, kidney, and liver homogenates were treated with ACN to precipitate proteins (1:1, *v*/*v*), centrifuged (2000× *g*, 2 min) and the supernatant was diluted with water (1:1, *v*/*v*). Urine was diluted 1:4 with water before injection.

### 2.6. Biodistribution and Tumor Uptake 

Preliminary biodistribution studies of ^177^Lu-labeled DOTA-MGS5, **1** and **2** at 4 h post injection (p.i.) were carried out in female BALB/c nude mice (Charles River Laboratories, Sulzfeld, Germany; n = 18) injected subcutaneously with A431-CCK2R cells, as well as A431-mock cells (2 × 10^6^ in 200 μL DMEM medium for each cell line) in the right and left flank at an age of 6–8 weeks. Then, after 7–11 days, when visible tumor xenografts were formed, groups of 5 mice were injected intravenously via a lateral tail vein with ~0.5 MBq of radiolabeled peptide, corresponding to 20 pmol of peptide, in a total volume of ~150 µL in PBS/0.5% BSA. Using an additional mouse per group, a blocking study was performed by co-injecting a 1000-fold molar excess of the respective peptide analog (20 nmol) together with the radiolabeled peptide. For quantification, a 1:1, 1:10 and 1:50 standard was prepared using aliquots of the injection solution mixed with PBS/0.5% BSA. Mice were euthanized by cervical dislocation 4 h after injection. Subsequently, a blood sample was collected, and different tissues were dissected. All samples, as well as the rest of the body were weighed, and the activity measured in the γ-counter together with the standard. Results were expressed as percentage of injected activity per gram of tissue (%IA/g).

Based on these preliminary studies, [^177^Lu]Lu-**2** was selected for further biodistribution studies evaluating the tumor uptake and tissue distribution for up to 7 days after injection. This study was approved by the Nijmegen Medical Center animal ethics committee (RUDEC) and the Dutch animal ethics committee (CCD) of the Radboud University (2020–0007-020). Female BALB/c nude mice were inoculated with A431-CCK2R cells at an age of 8–10 weeks (n = 20). When tumor xenografts were formed, mice were injected intravenously via a lateral tail vein with ~1 MBq of [^177^Lu]Lu-**2**, corresponding to 20 pmol of peptide, in a total volume of 200 µL in PBS/0.5% BSA. For quantification, a 1% standard was used. At different time points of 1 h, 24 h, 3 days, and 7 days after injecton, animals were euthanized by CO_2_/O_2_-asphyxiation and a blood sample was immediately drawn, and tissues of interest (spleen, pancreas, stomach, intestine, kidneys, liver, heart, lung, muscle, femur, A431-CCK2R tumor, and A431-mock tumor) were dissected. All collected samples were weighed and measured in the γ-counter together with the standard. Results were expressed as percentage of injected activity per gram of tissue (%IA/g), and tumor-to-organ activity ratios were calculated for selected tissues. For dose-limiting organs, kidneys and stomach, dosimetry estimates were calculated using Olinda-EXM (version 2.2, Vanderbilt University) and standard weights for humans (male and female). For this purpose, the % IA/g determined in mice was extrapolated to humans using the following equation:

$$\%\;\mathrm{IA}\;\mathrm{per}\;\mathrm{organ}\;\mathrm{in}\;\mathrm{humans}=\left[\%\mathrm{IA}/\mathrm{gin}\;\mathrm{mice}\times \mathrm{mass}\;\mathrm{of}\;\mathrm{mice}\;\left(\mathrm{kg}\right)\right]\times \left(\frac{\mathrm{mass}\;\mathrm{of}\;\mathrm{human}\;\mathrm{organ}\;\left(g\right)}{\mathrm{total}\;\mathrm{body}\;\mathrm{mass}\;\mathrm{of}\;\mathrm{human}\;\left(\mathrm{kg}\right)}\right)\;$$

Time scaling was additionally applied to account for the faster kinetics in mice versus humans using the following equation [25]:

$$\mathrm{time}\;\mathrm{in}\;\mathrm{humans}=\mathrm{time}\;\mathrm{in}\;\mathrm{mice}\times {\left[\frac{\mathrm{mass}\;\mathrm{of}\;\mathrm{human}\;\left(\mathrm{kg}\right)}{\mathrm{mass}\;\mathrm{of}\;\mathrm{mice}\;\left(\mathrm{kg}\right)}\right]}^{\frac{1}{4}}$$

The uptake values in mice were decay corrected for the adopted time points. 

## 3. Results

### 3.1. Peptide Synthesis

**Figure 1:**
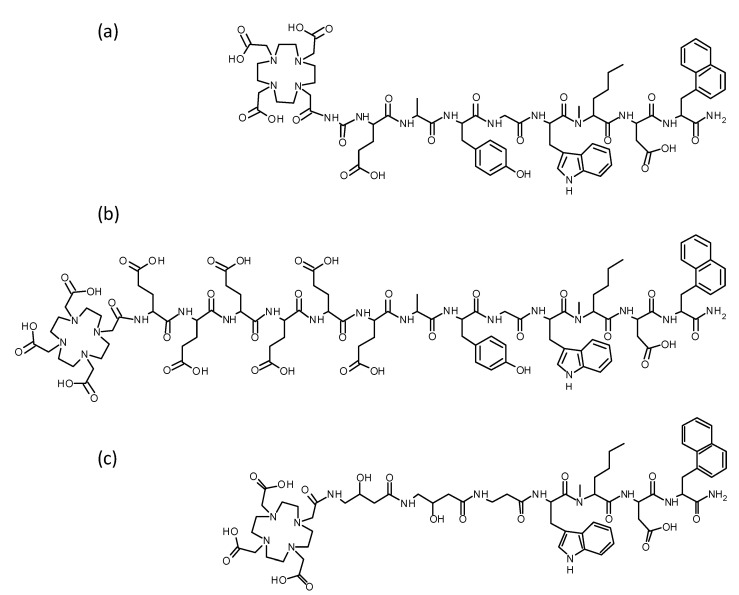
Chemical structure of (**a**) DOTA-MGS5, (**b**) **1** and (**c**) **2**.

The MG analogs **1** and **2** were synthesized starting with 100 mg resin, 240 µmol of each Fmoc-protected amino acid, and 140 µmol DOTA-tris(*tert*-butyl ester) according to a synthesis protocol published previously [24]. Following purification by RP-HPLC and characterization by MALDI-TOF MS, the peptides were obtained with a synthesis yield of <10% and chemical purity of ≥98%. The amino acid sequences and analytical data of the new peptide analogs are shown in Figure 1 and in Table 1. UV-chromatograms and mass spectrometry spectra are available in the Supplementary Materials (Figures S1 and S2). DOTA-MGS5 was obtained from piCHEM with confirmed purity of more than ≥97%.

### 3.2. Radiolabeling and Characterization In Vitro

Radiolabeling with lutetium-177 at an apparent molar activity of 10–15 GBq/µmol using the described standard protocol resulted in a RCP of ≥95%. Radiocolloid formation was ≤1%. For cell uptake studies, the reaction solutions were diluted with PBS. For stability and biodistribution studies, the radiopeptides were prepared at a higher apparent molar activity of 20–40 GBq/µmol also reaching RCP ≥95%. To avoid the presence of non-complexed lutetium-177 in these experiments, the reaction mixture was purified using a SepPak^®^ cartridge, as described above to remove hydrophilic impurities. Exemplary radiochromatograms after radiolabeling and after SPE purification are presented in the Supplementary Materials (see Figure S3). Only for metabolic studies with higher injected activities of ~37 MBq no purification was carried out to avoid the intravenous injection of higher amounts of ethanol.

**Figure 2:**
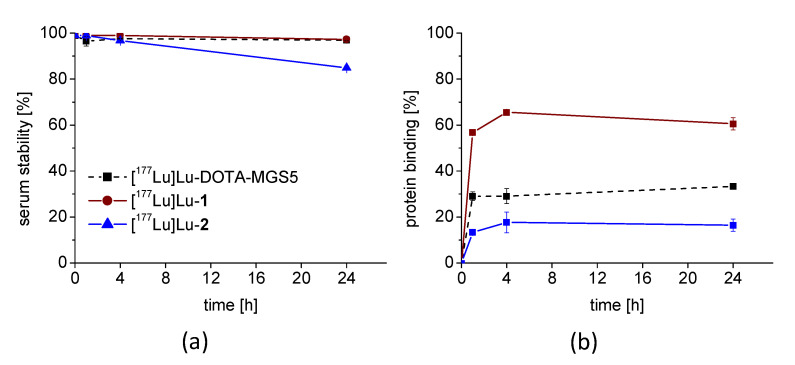
Serum stability (**a**) and protein binding (**b**) of the ^177^Lu-labeled peptide analogs, as determined by incubation in human serum at 37 °C up to 24 h.

The new radiopeptides exhibited variable stability in fresh human serum. [^177^Lu]Lu-**1** showed a high stability after 24 h incubation, comparable to [^177^Lu]Lu-DOTA-MGS5 (97.2 ± 0.1% and 96.9 ± 0.3%, respectively). For [^177^Lu]Lu-**2**, a significantly lower stability with only 84.8 ± 0.8% intact radiopeptide was observed after 24 h incubation (*p* < 0.05). The logD values in octanol/PBS showed the highest hydrophilicity for [^177^Lu]Lu-**1** (−4.18 ± 0.24) followed by [^177^Lu]Lu-DOTA-MGS5 (−2.25 ± 0.13) and [^177^Lu]Lu-**2** (−2.18 ± 0.38) with comparable values. Binding to human serum proteins was increased by a factor of two for [^177^Lu]Lu-**1**, whereas [^177^Lu]Lu-**2** showed a decrease in protein binding of ~50% when compared to [^177^Lu]Lu-DOTA-MGS5. The serum stability and protein binding over time is graphically shown in Figure 2 for all three radiopeptides.

### 3.3. Cell Uptake and Receptor Binding Studies

**Figure 3:**
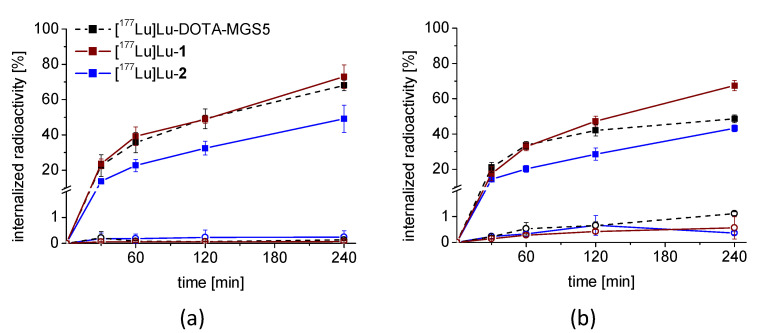
Cell uptake over time of [^177^Lu]Lu-DOTA-MGS5, [^177^Lu]Lu-**1** and [^177^Lu]Lu-**2** in (**a**) A431-CCK2R cells and in (**b**) AR42J rat pancreatic cells for up to 4 h after incubation. Uptake in A431-mock cells and blocking experiment in AR24J cells is additionally shown (circles). Values are expressed as mean ± SD from three independent experiments.

For all ^177^Lu-labeled peptide analogs, a high cell uptake was found for both CCK2R-expressing cell lines. [^177^Lu]Lu-**1** showed highest uptake in AR42J cells with uptake values of 67.5 ± 2.9% after 4 h of incubation, whereas for [^177^Lu]Lu-DOTA-MGS5 and [^177^Lu]Lu-**2** a somewhat lower uptake was observed at the same timepoint (48.6 ± 2.2% and 43.2 ± 1.9%, respectively). In A431-CCK2R cells, [^177^Lu]Lu-**1** showed even higher uptake values with 73.0 ± 6.6% after 4 h after incubation, whereas for [^177^Lu]Lu-DOTA-MGS5 and [^177^Lu]Lu-**2,** uptake values of 68.0 ± 3.0% and 49.2 ± 7.7% were found, respectively. The cell uptake in A431-mock cells without receptor expression, as well as in blocking experiments using pentagastrin for AR42J cells remained below ~1% for all ^177^Lu-labeled peptide analogs at any timepoint studied. In Figure 3, the cell uptake of the three radiopeptides over time is shown for both cell lines.

**Figure 4:**
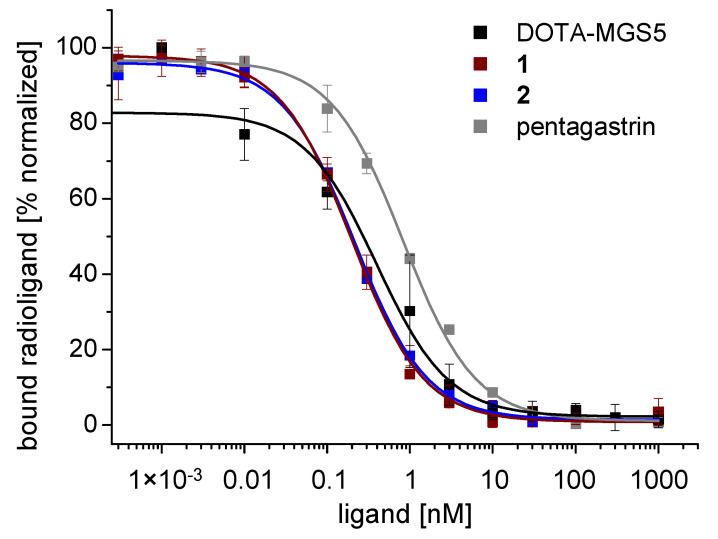
Exemplary normalized binding curves for DOTA-MGS5, **1**, **2** and pentagastrin in competition assays against [Leu^15^]gastrin-I radiolabeled with iodine-125 on A431-CCK2R cells.

For both peptide analogs, a high affinity to the CCK2R comparable to pentagastrin and DOTA-MGS5 was found. The lowest IC_50_ value of 0.18 ± 0.02 nM (n = 3) was found for **1**. For **2**, a slightly higher IC_50_ value of 0.24 ± 0.8 nM (n = 3) was calculated. However, almost overlapping binding curves were observed for the two peptide analogs. Both peptide analogs demonstrated a higher affinity to the CCK2R compared to the reference peptide pentagastrin (0.84 ± 0.22 nM; n = 3) and previously studied DOTA-MGS5 (0.4 ± 0.2; n = 3) [17]. Figure 4 shows exemplary normalized IC_50_ binding curves of **1** and **2** in comparison with pentagastrin and DOTA-MGS5. 

### 3.4. In Vivo Stability and Biodistribution Studies

**Figure 5:**
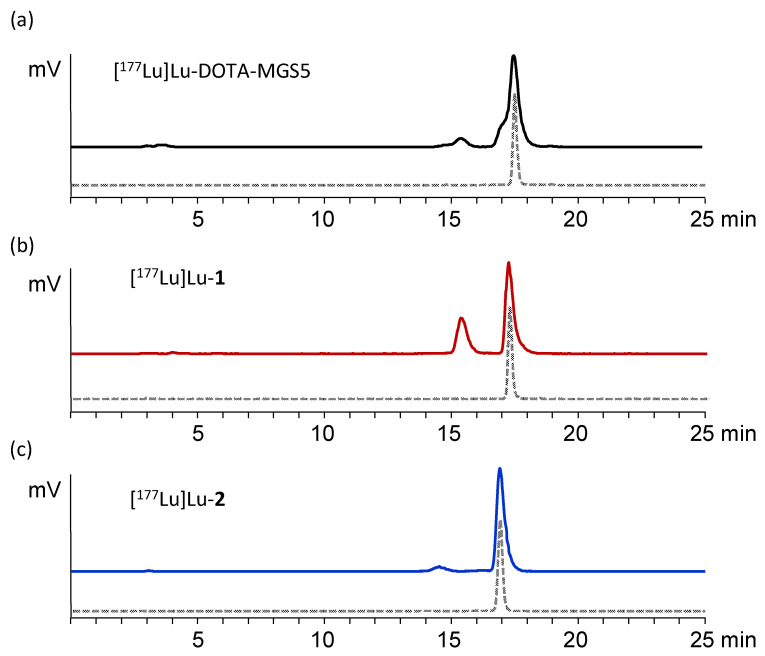
Radiochromatograms of the in vivo stability studies with (**a**) [^177^Lu]Lu-DOTA-MGS5, (**b**) [^177^Lu]Lu-**1**, and (**c**) [^177^Lu]Lu-**2** in BALB/c mice: colored lines showing analysis of blood samples 10 min p.i.; grey dotted line showing radiochromatogram after radiolabeling.

In the in vivo stability studies, a high resistance against enzymatic degradation could be observed for [^177^Lu]Lu-**2** when injected in BALB/c mice with more than 92.8 ± 0.76% and 84.4% intact radiopeptide in blood after 10 and 30 min after injection, respectively. A somewhat lower stability was found for [^177^Lu]Lu-**1** with values of 68.6 ± 2.3% and 44.0% for the same time points. Radiochromatograms of the blood samples at 10 min p.i. are displayed in Figure 5 in comparison with [^177^Lu]Lu-DOTA-MGS5 (86% intact radiopeptide) previously studied [17]. More pronounced enzymatic breakdown was found in the urine samples, with the highest amount of intact radiopeptide found for [^177^Lu]Lu-**2** (66.3 ± 3.0% and 60.2% after 10 and 30 min p.i., respectively). [^177^Lu]Lu-**1** showed a lower stability with only 42.4 ± 9.7% and 25.1% intact radiopeptide 10 and 30 min p.i., respectively. Furthermore, in kidney homogenates, a high rate of degradation was observed for [^177^Lu]Lu-**1** (17.5 ± 1.21% and 3.9% intact radiopeptide for 10 and 30 min, respectively), whereas [^177^Lu]Lu-**2** demonstrated a much higher amount of intact radiopeptide (59.7 ± 2.9% and 40.0% 10 and 30 min p.i., respectively). In liver tissue homogenate samples, 87.1 ± 18.27% and 78.1% intact radiopeptide was found for [^177^Lu]Lu-**2**, whereas [^177^Lu]Lu-**1** demonstrated only 54.9 ± 5.6% and 37.4% for the same time points.

**Figure 6:**
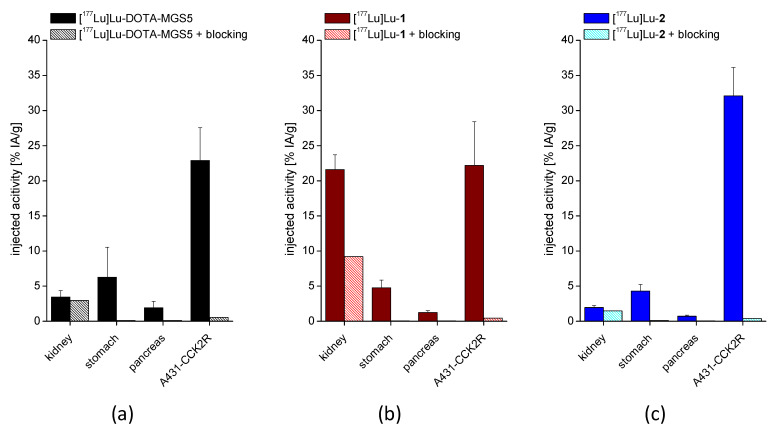
Uptake in kidney, stomach, pancreas, and A431-CCK2R xenograft of (**a**) [^177^Lu]Lu-DOTA-MGS5, (**b**) [^177^Lu]Lu-**1**, and (**c**) [^177^Lu]Lu-**2** at 4 h p.i. including blocking experiments using 1000-fold excess of non-radiolabeled peptide (n = 18).

In the biodistribution studies performed at 4 h p.i., all three ^177^Lu-labeled peptides showed a high accumulation of activity in A431-CCK2R xenografts with uptake values of 32.1 ± 4.1% IA/g for [^177^Lu]Lu-**2**, 22.2 ± 6.2% IA/g for [^177^Lu]Lu-**1** and 22.9 ± 4.7% IA/g for [^177^Lu]Lu-DOTA-MGS5. In A431-mock xenografts without CCK2R expression, a low uptake below 1% IA/g was found for all radiopeptides. Tumor weight at the time of sacrifice was 301 ± 153 mg for A431-CCK2R xenografts and 217 ± 207 mg for A431-mock xenografts (n = 24). In the blocking study performed with one single mouse for each radiopeptide, an effective inhibition of radioactivity accumulation in CCK2R-expressing tissue, with uptake values <1% IA/g in stomach, pancreas, and A431-CCK2R-xenografts could be confirmed for all ^177^Lu-labeled MG analogs. A very low tissue uptake below 1% IA/g was observed also for blood, heart, lung, muscle, and bone. Only the uptake of [^177^Lu]Lu-DOTA-MGS5 in the liver was slightly higher (1.02 ± 0.23% IA/g). [^177^Lu]Lu-**1** showed a considerably higher kidney uptake (21.6 ± 2.11% IA/g), whereas the kidney uptake of [^177^Lu]Lu-**2** (1.96 ± 0.29% IA/g) was reduced when compared to [^177^Lu]Lu-DOTA-MGS5 (3.45 ± 0.91% IA/g). In addition to that, the uptake of [^177^Lu]Lu-**1** in liver and spleen was also increased compared to both the other radiopeptides. In Figure 6, the uptake values found for A431-CCK2R xenografts and selected tissues (kidney, stomach, pancreas), including blocking, are graphically presented for all three radiopeptides. All other results from the biodistribution study are given in the Supplementary Materials (Tables S1 and S2). 

[^177^Lu]Lu-**2** showed the lowest uptake in dose-limiting organs, stomach and kidneys, while having the highest tumor accumulation. For this reason, this radiotracer was selected for an additional biodistribution study evaluating the tumor uptake and tissue distribution in A431-CCK2R xenografted BALB/c nude mice over up to seven days.

A rapid washout of the radioactivity from the blood pool was observed over time together with a low accumulation of radioactivity in most of the tissues. The uptake values in blood decreased from 1.45 ± 0.30% at 1 h p.i. to 0.03 ± 0.01% IA/g at 24 h p.i., and dropped to almost undetectable levels for the rest of the study. A low receptor-specific uptake in stomach was confirmed with values of 5.43 ± 1.05% IA/g at 1 h p.i. which was reduced by ~33% at 24 p.i. (3.66 ± 0.38% IA/g) and slowly decreased to 2.28 ± 0.33% and 0.88 ± 0.12% IA/g after 3 and 7 days, respectively. A remarkable low uptake in kidneys already at 1 h p.i. with uptake values of only 2.84 ± 0.42% IA/g was detected, which considerably decreased by ~54% after 24 h (1.32 ± 0.23% IA/g) and further declined below 1% IA/g at 3 and 7 days after injection. The uptake in A431-CCK2R xenografts with considerably high values of 56.29 ± 9.14% IA/g for the early time point of 1 h p.i., dropped by ~40% after 24 h (33.61 ± 2.95% IA/g) and continued to fall to levels of 12.52 ± 0.96% IA/g and 1.23 ± 0.53% IA/g 3 and 7 days after injection, respectively. Tumor weights, as determined at the time of sacrifice, were 174 ± 70 mg at 1 h p.i., 281 ± 66 mg at 24 h, 440 ± 73 mg at 3 days and 380 ± 258 mg 7 days after injection.

**Figure 7:**
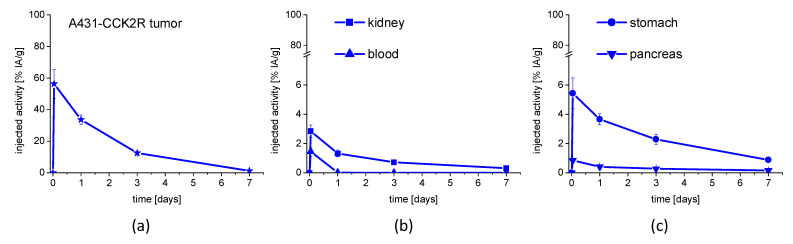
Uptake values of [^177^Lu]Lu-**2** in (**a**) A431-CCK2R tumor xenograft, (**b**) blood and kidney, and (**c**) stomach and pancreas for up to 7 days after injection (n = 5).

In Figure 7, the washout over time for selected tissues (A431-CCK2R xenograft, blood, kidney, stomach, and pancreas) is illustrated. Tumor-to-organ ratios for blood, stomach, and kidney are shown in Table 2. In the Supplementary Materials, the distribution over time in the remaining tissues is graphically shown and the uptake values for all tissues and time points analyzed are given (Figure S4 and Table S3).

Biodistribution data in A431-CCK2R xenografted BALB/c mice identified stomach and kidneys as the dose-limiting organs for PRRT. Extrapolation of the % IA per organ in humans was based on the mean body weight of mice (18.1 g, n = 20, range from 15.5–20.7 g) and the standard weights deduced from Olinda for men (body weight: 73.0 kg; stomach wall: 150 g, kidneys: 310 g) and women (body weight: 60.0 kg; stomach wall: 140 g, kidneys: 275.5 g). For time scaling, a factor of 7.5–8.0 was calculated from the mean body weight of mice and set to 10. The calculated organ dose for stomach, considering the wall as radiation source was 0.193 mGy/MBq for males and 0.2390 mGy/MBq for females. The calculated organ dose for kidneys was 0.0822 mGy/MBq for males and 0.0990 mGy/MBq for females. 

## 4. Discussion

Using naturally occurring ligands with a high affinity for a particular target receptor as molecular templates is a common strategy in the development of radiotracers in nuclear medicine. Typically, peptide-based radiotracers are coupled to bifunctional chelators enabling for a simple and fast radiolabeling process with high yields of associated radiometals, making them effective for SPECT, PET, and targeted radiotherapy. Several approaches have been explored to overcome the low stability in vivo or excessive accumulation in non-target tissue of radiolabeled MG analogs targeting CCK2R [6,26,27,28,29,30]. In the past, no other modification within the receptor-binding sequence “Trp-Met-Asp-Phe-NH_2_” than substitution of Met has been investigated in order to avoid a negative effect on the receptor affinity. The MG analog with the sequence DOTA-DGlu-Ala-Tyr-Gly-Trp-(*N*-Me)Nle-Asp-1Nal-NH_2_ (DOTA-MGS5) recently developed by our group showed favorable properties in terms of stabilization against metabolic degradation in vivo and enhanced tumor targeting. In this MG analog, site-specific modifications in the receptor-specific part of the linear peptide have been applied, by replacing Met with (*N*-Me)Nle, and Phe with 1Nal [17]. Additional replacement within the *N*-terminal peptide sequence of different amino acids with the cyclic amino acid proline did not lead to a further improvement ofthe stability in vivo [18]. Thus, in this study we have investigated alternative possibilities to change the *N*-terminal peptide sequence. It has been previously shown, that the penta-DGlu sequence in DOTA-PP-F11N improved the overall stability in vivo resulting in enhanced tumor uptake [28]. The insertion of the penta-DGlu sequence in DOTA-MGS5 as evaluated for [^177^Lu]Lu-**1**, resulted in higher hydrophilicity (−4.18 ± 0.24), however increased protein binding (~60% after 24 h incubation) was observed in human serum. In [^177^Lu]Lu-**2**, based on the sequence of pentagastrin, two moieties of GABOB were introduced as a linker, to improve the hydrophilic character of the conjugate. Previous studies with CCK4 derivatives confirmed the necessity to introduce a spacer, such as βAla or Ahx, between the bulky chelator and the receptor-specific sequence [20,21]. Furthermore, a favorable impact on renal absorption and stability against enzymatic degradation was shown for the introduction of non-charged amino acids, such as glutamine [30]. Even though similar logD values were observed for [^177^Lu]Lu-**2** and [^177^Lu]Lu-DOTA-MGS5, the applied modifications led to a 2-fold decrease in serum protein binding (16.5 ± 2.7% versus 33.3 ± 0.9%, respectively). The observed differences in protein binding might have affected the results of the stability study in human serum, as contrasting results were observed in the metabolic study in vivo. It has been shown previously, that in vitro studies are not sufficient to predict the stability of MG analogs against metabolic degradation [31]. The changes applied within the *N*-terminal peptide sequence did not negatively affect the receptor affinity of the peptide analogs. No additional binding assays were performed for the metal-complexes to investigate a possible influence of the metal on the receptor affinity. In previous studies with DOTA-MGS5, no considerable impact on the cell uptake and targeting properties could be observed when using different radiometals [17]. Only minor differences in the cell internalization were observed for all three ^177^Lu-labeled peptides in both CCK2R-expressing cell lines. For both peptide analogs, a low nanomolar receptor affinity compared to the standard peptide pentagastrin and DOTA-MGS5 previously studied was observed. A high cell uptake of 43–73% of the ^177^Lu-labeled peptides was confirmed for A431-CCK2R and AR42J cells at 4 h after incubation.

Most of the metabolic studies in vivo evaluating the stability of radiolabeled MG in the blood of mice have only been undertaken for short time periods of 5–10 min after injection. Given the improved stability of [^177^Lu]Lu-DOTA-MGS5, in this study also a later timepoint of 30 min after injection was examined to gain a better understanding of the stability of the novel MG analogs. Contrary to in vitro stability studies in human serum, [^177^Lu]Lu-**2** showed the highest in vivo stability with more than 84% intact radiopeptide after 30 min p.i., observed in the blood of BALB/c mice. A slightly lower stability of 77% was observed for to the lead structure [^177^Lu]Lu-DOTA-MGS5 [17]. [^177^Lu]Lu-**1** showed a lower in vivo stability with 44% intact radiopeptide detected in the blood of BALB/c mice at the same time point. Still, the in vivo stability is highly improved when compared to ^177^Lu-labelled CP04, for which only ~5% intact radiopeptide were observed for the same time point p.i. in previous studies [17].

The high stabilization of [^177^Lu]Lu-**2** against enzymatic degradation was connected with improved tumor targeting, whereas the observed minor differences in in vivo stability of [^177^Lu]Lu-**1** and [^177^Lu]Lu-DOTA-MGS5 did not impact the tumor targeting. Biodistribution studies were performed in A431-CCK2R and A431-mock xenografted BALB/c nude mice, allowing for a comparison with the results obtained for other CCK2R targeting peptide analogs. No human cell line with physiological CCK2R expression is currently available. In this study, a high uptake in A431-CCK2R xenografts of 22.9 ± 4.7 % IA/g was confirmed for [^177^Lu]Lu-DOTA-MGS5 at 4 h after injection. With the two new MG analogs with different modifications within the *N*-terminal peptide sequence, a tumor uptake of 22.2 ± 6.2% IA/g was observed for [^177^Lu]Lu-**1** and of 32.1 ± 4.1% IA/g for [^177^Lu]Lu-**2**. The low uptake in A431-mock xenografts and the additional blocking experiment clearly confirmed the specificity of the CCK2R-mediated uptake. All three radiopeptides showed low accumulation in non-target tissue. However, the kidney uptake of [^177^Lu]Lu-**1** was ~6 times higher when compared to [^177^Lu]Lu-DOTA-MGS5, confirming that the introduction of negatively charged *N*-terminal amino acids affects the accumulation in renal tissue [30]. A much lower kidney uptake was observed for [^177^Lu]Lu-**2**, which resulted to be decreased by a factor of ~2 in comparison with [^177^Lu]Lu-DOTA-MGS5. Shortening of the amino acid sequence and introduction of a non-charged hydrophilic linker led to a considerable reduction in kidney uptake, resulting in a more than 2-fold improvement in tumor-to-kidney ratio (17 vs. 7, respectively).

For application in PRRT, increasing the radiation dose to the tumor cell while simultaneously decreasing the absorbed dose to non-target tissues is of the utmost importance. When looking at the biodistribution profile of [^177^Lu]Lu-**2** over time, a high and persistent tumor uptake was achieved. A somewhat faster washout of the radioactivity from A431-CCK2R xenografts with values of ~56% versus ~13% IA/g at 1 h and 3 days after injection was observed as compared to CCK2R-expressing stomach. However, tumor-to-organ ratio at 3 days p.i. was still ~6 for stomach and ~17 for kidneys. Thus, with [^177^Lu]Lu-**2**, showing a tumor uptake of >30% IA/g up to 24 h p.i., a 4-fold improvement in tumor uptake in comparison with [^177^Lu]Lu-PP-F11N, currently under clinical investigation, could be achieved [19,28]. The high tumor uptake of [^177^Lu]Lu-**2**, combined with low accumulation in other tissues, resulted in very favorable tumor-to-organ ratios, also including stomach and kidneys, which have been identified as dose-limiting organs. Radiation-induced damage to the dose-limiting organs is reduced during PRRT by dividing the radioactivity to be administered into multiple cycles. By extrapolation of the absorbed dose in the stomach wall and kidneys from mouse to humans and considering four consecutive treatments with 8 GBq of [^177^Lu]Lu-**2**, a cumulative absorbed radiation dose of <10 and <5 Gy was estimated for stomach and kidneys, respectively. Thus it can be expected, that during PRRT, the cumulative doses to stomach (50 Gy) and kidneys (27 Gy) will not be reached [32,33]. These values compare well to the previous published data on dosimetry calculations for PRRT with [^177^Lu]Lu-PP-F11N in patients, anticipating a cumulative dose of <15 Gy for the stomach and <5 Gy for the kidneys after four cycles with 8 GBq [19].

## 5. Conclusions

In this study, two new ^177^Lu-labeled MG analogs based on the sequence of DOTA-MGS5 by either introduction of a penta-DGlu moiety or depletion of the four *N*-terminal amino acids combined with introduction of GABOB-GABOB-βAla as a linker were synthesized and evaluated for their potential therapeutic use. The introduction of multiple negative charges into [^177^Lu]Lu-**1** clearly affected renal accumulation leading to a suboptimal biodistribution profile, whereas the combined use of the receptor-specific sequence “Trp-(*N*-Me)Nle-Asp-1Nal-NH_2_”of DOTA-MGS5 and a non-charged hydrophilic linker in [^177^Lu]Lu-**2** led to a further enhancement in tumor uptake and favorable tumor-to-organ ratios, confirming the high potential of this new class of radiolabeled MG analogs for therapeutic use in CCK2R-expressing malignancies.

## Figures and Tables

**Table 1:** Amino acid sequence and analytical data of DOTA-MGS5, **1** and **2**.

Peptide	Amino Acid Sequence	Purity	MW Calc *m*/*z* [M + H]^+^	MW Found *m*/*z* [M + H]^+^
DOTA-MGS5	DOTA-DGlu-Ala-Tyr-Gly-Trp-(*N*-Me)Nle-Asp-1Nal-NH_2_	>95%	1449.67	1450.20
1	DOTA-(DGlu)_6_-Ala-Tyr-Gly-Trp-(*N*-Me)Nle-Asp-1Nal-NH_2_	≥98%	2095.13	2094.13
2	DOTA-(GABOB)_2_-βAla-Trp-(*N*-Me)Nle-Asp-1Nal-NH_2_	≥98%	1302.43	1302.34

**Table 2:** Tumor-to-organ ratios of [^177^Lu]Lu-**2** in A431-CCK2R xenografted BALB/c nude mice at 1 h, 24 h, 3 days, and 7 days after injection.

Time Point p.i.	1 h	24 h	3 Days	7 Days
Tumor-to-blood	38.9	3240.4	4856.5	241.2
Tumor-to-stomach	10.4	9.2	5.5	1.4
Tumor-to-kidney	19.8	25.5	17.3	4.0

## Data Availability

Data are contained within the article.

## Supplementary Materials

The following supporting information can be downloaded at: https://www.mdpi.com/article/10.3390/pharmaceutics15030796/s1, Figure S1: UV-chromatograms of (a) DOTA-MGS5, (b) 1, and (c) 2 after HPLC purification; Figure S2: Mass spectra of (a) DOTA-MGS5, (b) 1, and (c) 2 after HPLC purification; Figure S3: Radiochromatograms of ^177^Lu-labeled (a) DOTA-MGS5, (b) 1, and (c) 2: reaction mixture after radiolabeling with minor presence (≤2%) of free lutetium-177 (solid line) and after removal of hydrophilic impurities by solid phase extraction (dashed line), respectively; Table S1: Results of biodistribution studies evaluated in A431-CCK2R/A431-mock xenografted BALB/c nude mice of the ^177^Lu-labeled peptide derivatives (~0.5 MBq, 20 pmol, 4 h p.i.). Values expressed as percentage of injected activity per gram tissue (% IA/g); mean ± SD (n = 5); Table S2: Results of the additional biodistribution study with 1000-fold excess of non-radiolabeled peptide (blocking experiment) in A431-CCK2R/A431-mock xenografted BALB/c nude mice of the ^177^Lu-labeled peptide derivatives (~0.5 MBq, 20 pmol, 4 h p.i.). Values expressed as percentage of injected activity per gram tissue (% IA/g; n = 1); Figure S4: Uptake of [^177^Lu]Lu-2 in different tissues dissected from A431-CCK2R xenografted BALB/c nude mice for up to 7 days after injection (n = 5); Table S3: Uptake values of [^177^Lu]Lu-2 in A431-CCK2R tumor xenograft for up to 7 days after injection (n = 5).
