# Supplementary material for: Effect of N-Terminal Peptide Modifications on In Vitro and In Vivo Properties of 177Lu-Labeled Peptide Analogs Targeting CCK2R
Source: Pharmaceutics. 2023 Feb 28;15(3):796. doi: 10.3390/pharmaceutics15030796 (PMC10058949; doi:10.3390/pharmaceutics15030796)
Supplement: Supplementary file 1 [file pharmaceutics-15-00796-s001.zip › pharmaceutics-2160165-supplementary.pdf]

Table of content

Figure S1. UV-chromatograms of (a) DOTA-MGS5, (b) 1 and (c) 2 after HPLC purification.

Figure S2. Mass spectra of (a) DOTA-MGS5, (b) 1 and (c) 2 after HPLC purification.

Figure S3. Radiochromatograms of ^{177}Lu-labeled (a) DOTA-MGS5, (b) 1 and (c) 2: reaction mixture after radiolabeling with minor presence (≤2%) of free lutetium-177 (solid line) and after removal of hydrophilic impurities by solid phase extraction (dashed line), respectively.

Table S1. Results of biodistribution studies evaluated in A431-CCK2R/A431-mock xenografted BALB/c nude mice of the ^{177}Lu-labeled peptide derivatives (~0.5 MBq, 20 pmol, 4 h p.i.). Values expressed as percentage of injected activity per gram tissue (% IA/g); mean ± SD (n=5).

Table S2. Results of the additional biodistribution study with 1000-fold excess of non-radiolabeled peptide (blocking experiment) in A431-CCK2R/A431-mock xenografted BALB/c nude mice of the ^{177}Lu-labeled peptide derivatives (~0.5 MBq, 20 pmol, 4 h p.i.). Values expressed as percentage of injected activity per gram tissue (% IA/g; n=1).

Figure S4. Uptake of [^{177}Lu]Lu-2 in different tissues dissected from A431-CCK2R xenografted BALB/c nude mice for up to 7 days after injection (n=5).

Table S3. Uptake values of [^{177}Lu]Lu-2 in A431-CCK2R tumor xenograft for up to 7 days after injection (n=5).

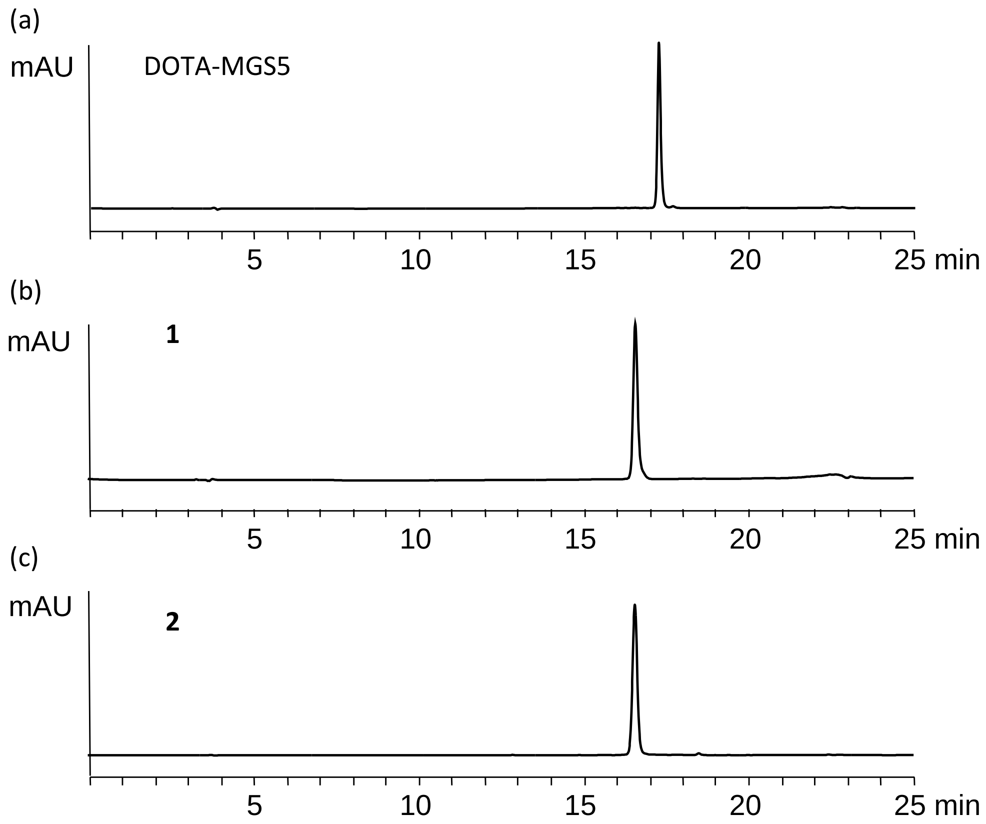

Figure S1. UV-chromatograms of (a) DOTA-MGS5, (b) **1** and (c) **2** after HPLC purification.

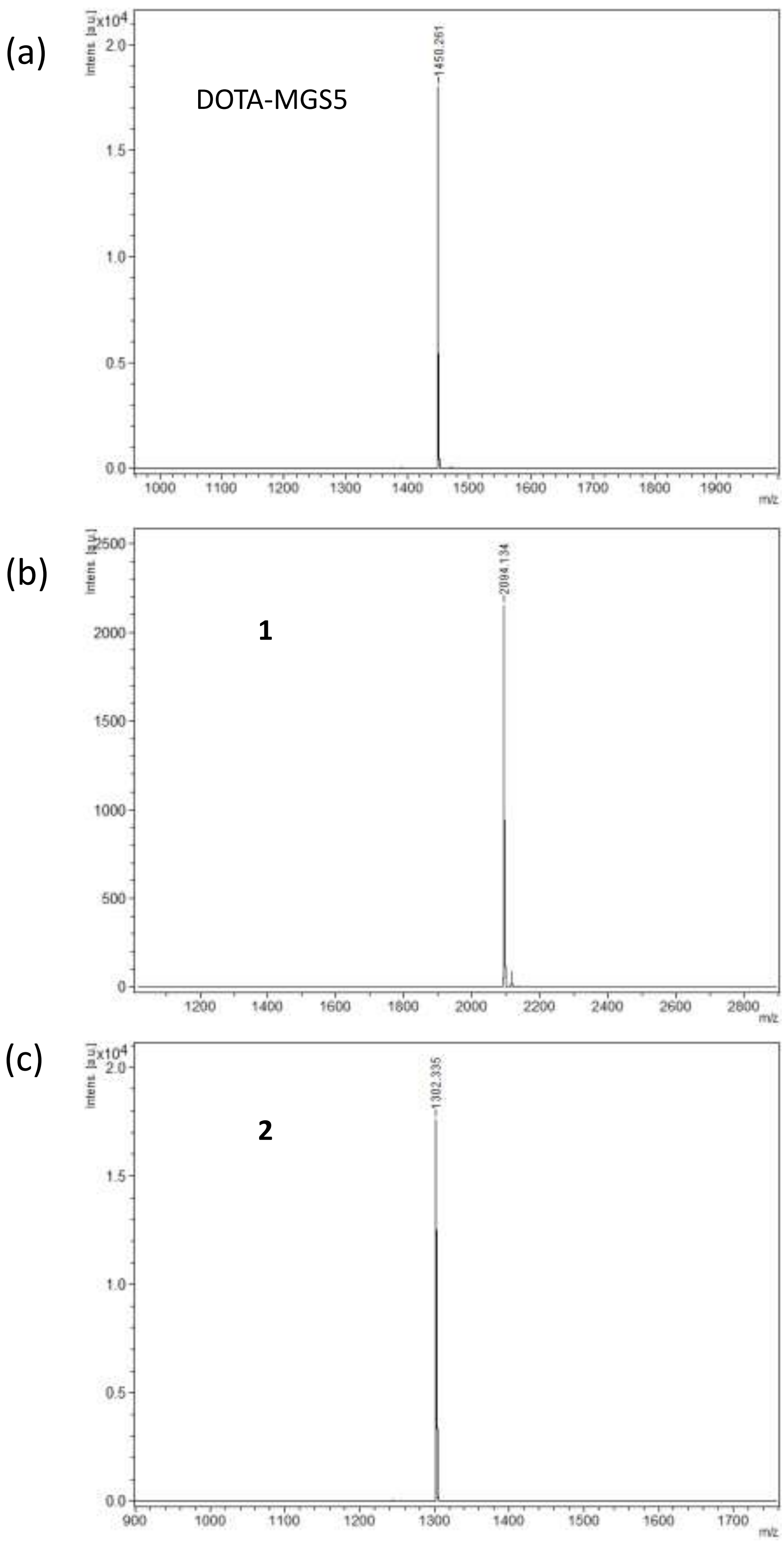

Figure S2. Mass spectra of (a) DOTA-MGS5, (b) **1** and (c) **2** after HPLC purification

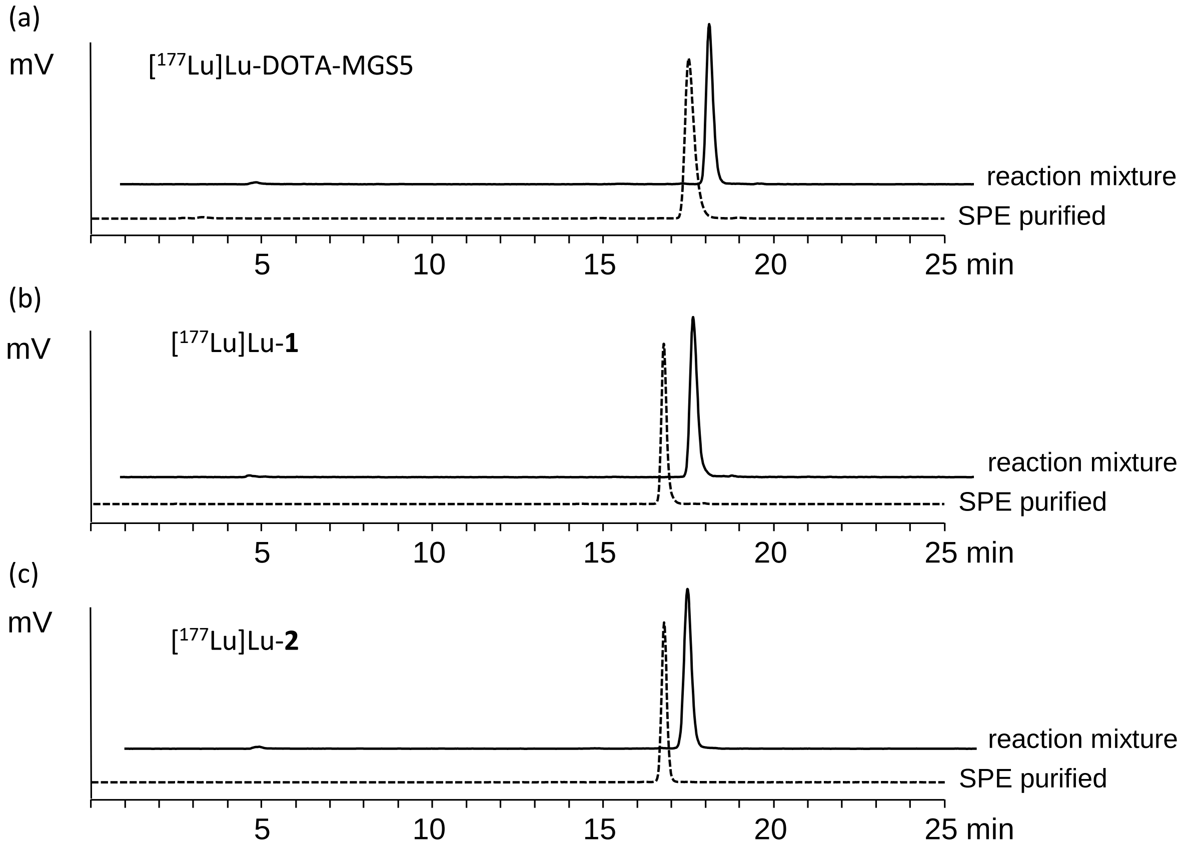

Figure S3. Radiochromatograms of ^{177}Lu-labeled (a) DOTA-MGS5, (b) **1** and (c) **2**: reaction mixture after radiolabeling with minor presence (≤2%) of free lutetium-177 (solid line) and after removal of hydrophilic impurities by solid phase extraction (dashed line), respectively.

Table S1. Results of biodistribution studies evaluated in A431-CCK2R/A431-mock xenografted BALB/c nude mice of the ^{177}Lu-labeled peptide derivatives (~0.5 MBq, 20 pmol, 4 h p.i.). Values expressed as percentage of injected activity per gram tissue (% IA/g); mean ± SD (n=5).

radiopeptide	[^{177}Lu]Lu-DOTA-MGS5	[^{177}Lu]Lu-1	[^{177}Lu]Lu-2
**blood**	0.11±0.11	0.02±0.00	0.03±0.01
**lung**	0.17±0.14	0.12 ±0.04	0.22±0.06
**heart**	0.10±0.06	0.06±0.01	0.07±0.02
**femur**	0.36±0.35	0.21±0.05	0.22±0.15
**muscle**	0.10±0.02	0.04±0.02	0.06±0.03
**spleen**	0.27±0.18	1.86±0.62	0.96±0.30
**intestine**	1.02±0.23	0.42±0.07	0.60±0.12
**liver**	1.02±0.80	2.48±0.39	1.43±0.18
**kidneys**	3.45±0.91	21.60±2.11	1.96±0.29
**stomach**	6.26±4.28	4.77±1.10	4.30±0.95
**pancreas**	1.91±0.91	1.25±0.27	0.72±0.14
**A431-CCK2R**	22.8±4.67	22.18±6.23	32.10±4.05
**A431-mock**	0.19±0.03	0.14±0.04	0.22±0.06

Table S2. Results of the additional biodistribution study with 1000-fold excess of non-radiolabeled peptide (blocking experiment) in A431-CCK2R/A431-mock xenografted BALB/c nude mice of the ^{177}Lu-labeled peptide derivatives (~0.5 MBq, 20 pmol, 4 h p.i.). Values expressed as percentage of injected activity per gram tissue (% IA/g; n=1).

radiopeptide	[^{177}Lu]Lu-DOTA-MGS5	[^{177}Lu]Lu-1	[^{177}Lu]Lu-2
**blood**	0.09	0.03	0.02
**lung**	0.08	0.06	0.20
**heart**	0.08	0.05	0.04
**femur**	0.04	0.18	0.09
**muscle**	0.03	0.02	0.04
**spleen**	0.16	1.13	0.70
**intestine**	0.79	0.17	0.45
**liver**	0.54	2.86	1.13
**kidneys**	2.95	9.21	1.47
**stomach**	0.09	0.06	0.09
**pancreas**	0.11	0.05	0.05
**A431-CCK2R**	0.54	0.45	0.39
**A431-mock**	0.34	0.12	0.16

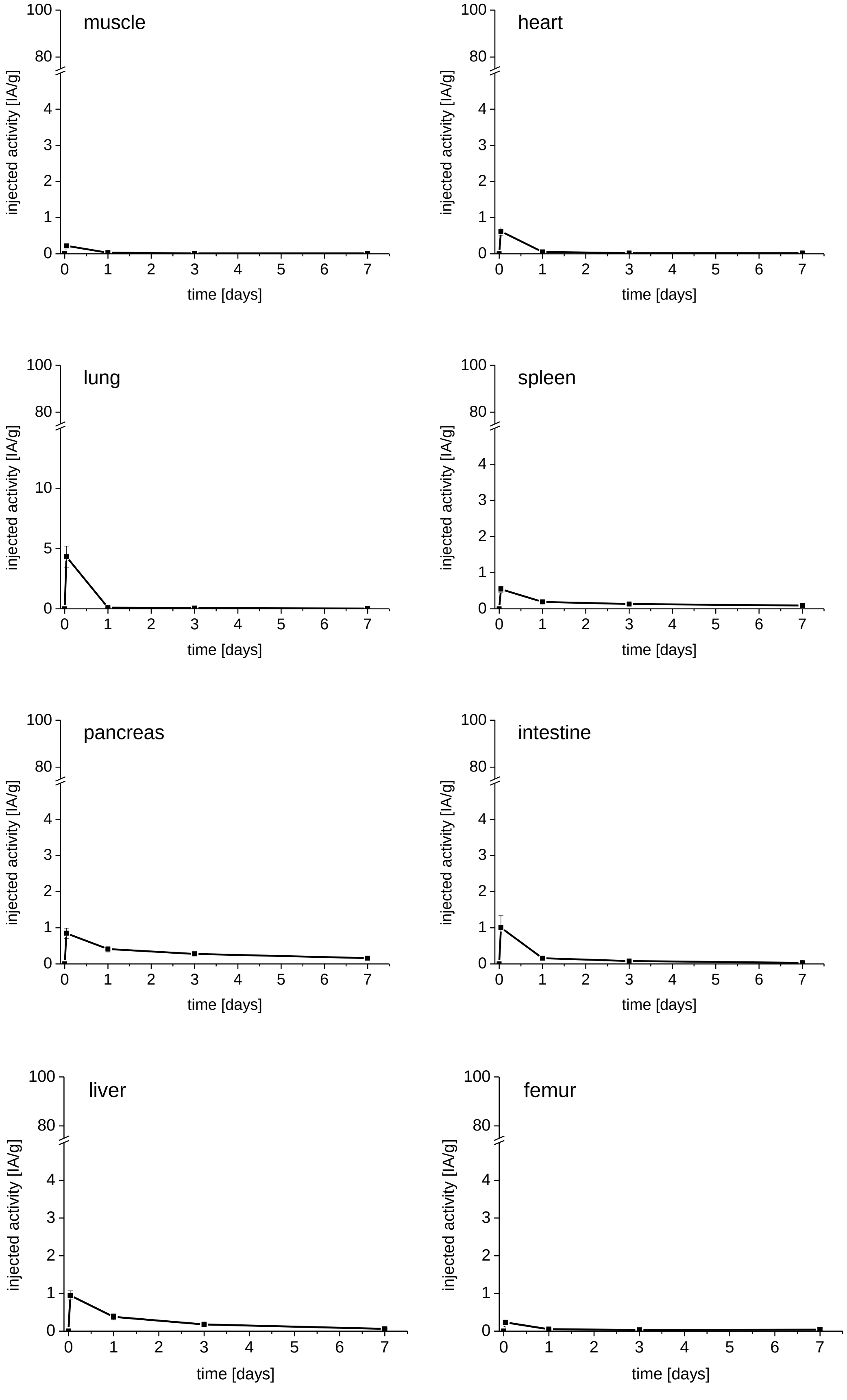

Figure S3. Uptake of [^{177}Lu]Lu-**2** in different tissues dissected from A431-CCK2R xenografted BALB/c nude mice for up to 7 days after injection (n=5).

Table S3. Uptake values of [^{177}Lu]Lu-**2** in A431-CCK2R tumor xenograft for up to 7 days after injection (n=5).

**Timepoint p.i.**	**1 h** [% IA/g]	**24 h** [% IA/g]	**3 days** [% IA/g]	**7 days** [% IA/g]
**blood**	1.45±0.30	0.01±0.00	0.00±0.00	0.01±0.00
**lung**	4.33±0.87	0.09±0.02	0.06±0.01	0.02±0.01
**heart**	0.62±0.12	0.05±0.01	0.02±0.00	0.02±0.00
**femur**	0.23±0.05	0.05±0.01	0.03±0.01	0.04±0.02
**muscle**	0.22±0.05	0.03±0.00	0.01±0.00	0.01±0.01
**spleen**	0.54±0.08	0.19±0.03	0.13±0.02	0.09±0.04
**intestine**	1.00±0.34	0.16±0.02	0.08±0.00	0.03±0.00
**liver**	0.95±0.12	0.38±0.08	0.18±0.01	0.06±0.01
**kidneys**	2.84±0.42	1.32±0.23	0.72±0.10	0.31±0.03
**stomach**	5.43±1.05	3.66±0.38	2.28±0.33	0.88±0.12
**pancreas**	0.85±0.14	0.41±0.08	0.28±0.04	0.16±0.04
**A431-CCK2R**	56.29±9.14	33.61±2.95	12.52±0.96	1.23±0.53
